# Correlation Between Early Arterial Lactate Levels, Arterial Bicarbonate Ion Levels, and the Lactate/Albumin Ratio and In‐Hospital Mortality in Patients With Acute Myocardial Infarction Complicated With Cardiac Shock

**DOI:** 10.1155/crp/9995508

**Published:** 2026-04-06

**Authors:** Jiao Wang, Meijuan Zheng, Yuchun Yang, Zhen Bao, Muhuyati Wulasihan

**Affiliations:** ^1^ Department of Integrated Cardiology, First Affiliated Hospital of Xinjiang Medical University, Urumqi, Xinjiang, China, xjmu.edu.cn; ^2^ Key Laboratory of Special Environment and Health Research in Xinjiang, Urumqi, Xinjiang, China; ^3^ Xinjiang Medical University, Urumqi, Xinjiang, China, xjmu.edu.cn

**Keywords:** acute myocardial infarction with cardiogenic shock (AMICS), arterial bicarbonate ion, hospital mortality, lactate/albumin ratio (L/A)

## Abstract

**Background:**

To investigate the connection between early arterial lactate, arterial bicarbonate ion, lactate/albumin ratio (L/A) and in‐hospital mortality in patients with acute myocardial infarction complicated with cardiac shock (AMICS).

**Methods:**

A receiver operating characteristic (ROC) curve was drawn to analyze the predictive value of these indicators for in‐hospital mortality in 395 patients with AMICS. A Kaplan‒Meier survival curve was drawn to analyze the median survival time of each subgroup. A Cox fitted proportional risk model was used to verify the association between these indicators and all‐cause mortality during hospitalization.

**Results:**

ROC curve analysis revealed that the areas under the curve for arterial bicarbonate ion level, arterial lactate level, and L/A were 0.707, 0.679, and 0.670, respectively, indicating that these parameters have certain value for predicting in‐hospital mortality in patients with AMICS. The Kaplan‒Meier survival curve revealed that the median survival time of the low bicarbonate ion group was shorter than that of the high bicarbonate ion group, and the median survival time of the low lactic acid group and low L/A group was longer than that of the high lactic acid group and the high L/A group. The Cox proportional risk model indicated that higher arterial bicarbonate ion levels were a protective factor for in‐hospital death in AMICS patients and that higher arterial lactate levels and a higher L/A were independent risk factors.

**Conclusion:**

Early arterial bicarbonate ion levels, arterial lactate levels, and the L/A were predictive of all‐cause death in AMICS patients.

## 1. Introduction

Acute myocardial infarction (AMI) is the most common cause of cardiogenic shock (CS), and CS is also the most common serious complication and cause of death in AMI patients [1]. According to the global research registry, the overall incidence of acute myocardial infarction with cardiogenic shock (AMICS) ranges from 4% to 12%; however, modern interventional surgery has substantially improved the revascularization rate in early AMI patients. However, the 30‐day mortality rate is still as high as 40%–45% [2–4]. In 2022, 1034 million AMI patients were hospitalized in China, 7.8% of whom were also diagnosed with cardiac shock [5]. Moreover, in previous studies, researchers reported that advanced age, pulmonary infection, prior cerebral infarction, and chronic renal insufficiency are risk factors for AMICS [6].

Lactic acid is a product of anaerobic metabolism as well as the prediction of the prognosis of critical diseases and the severity of shock [7]. It has been shown to be associated with mortality from multiple critical illnesses, acute heart failure, out‐of‐hospital cardiac arrest, and other diseases [8–10]. A study of 1176 acute ST‐elevation myocardial infarction (STEMI) patients revealed that elevated lactate levels were associated with increased risks of 1‐day acute mortality and 30‐day mortality [11]. Other studies have revealed that blood lactic acid is an independent predictor of in‐hospital mortality in STEMI patients with CS [12]. The concentration of bicarbonate ions in arterial blood reflects the acid‒base metabolism of the body. Wigger et al. conducted a large cohort study of visceral shock patients in the ICU and reported that a lower serum bicarbonate level at admission was an independent predictor of CS‐related mortality [13]. In other studies, researchers suggest that a low serum bicarbonate level at admission is a significant predictor of mortality in patients with acute aortic dissection [14]. Several studies have revealed that serum albumin levels are associated with cardiovascular disease. He et al. reported that a low serum albumin level is an independent risk factor for atrial fibrillation [15, 16]. Other studies [17, 18] have suggested that the serum albumin level is an independent risk factor for AMI in the Chinese Han population and that a low serum albumin level can increase the risk of long‐term all‐cause mortality in AMI patients.

All the above single indices have certain predictive value for the prognosis of AMI. Although the lactate/albumin ratio (L/A) has recently been proposed as a complex indicator, its use has been limited to sepsis [19, 20], heart failure [21], and other diseases in most studies. Other studies have revealed that the L/A has predictive value for 30‐day mortality in AMI patients [22] and that the L/A has independent predictive ability for all‐cause mortality in AMI patients during hospitalization [23]. However, the sample of patients included in the above studies was derived from the MIMIC database, which does not include the Chinese population, and the study population was not limited to AMICS patients. At present, the predictive value of the L/A, acid–base balance, and other related indicators in the AMICS population lacks theoretical support. Therefore, adult AMICS patients in the Xinjiang region of China were selected for analysis of the predictive value of early arterial lactate levels, arterial bicarbonate ion concentration, and the L/A for all‐cause death during the hospital stay.

## 2. Methods

### 2.1. Study Participants

The retrospective study was approved by the Medical Ethics Committee of the First Affiliated Hospital of Xinjiang Medical University (approval No. K202412‐25) and was conducted in accordance with the Declaration of Helsinki and its amendments. A total of 395 patients with AMI complicated with CS hospitalized at the First Affiliated Hospital of Xinjiang Medical University between January 2018 and August 2024 were included in this retrospective study, of whom 171 died during their hospital stay and 224 were discharged because their condition had improved, and the mortality rate was 43.29%.

#### 2.1.1. Inclusion Criteria

The inclusion criteria include the following: (1) Patients aged 18 years and older diagnosed with AMICS on the basis of the diagnostic criteria put forward by the Chinese Expert Consensus on the Diagnosis and Treatment of Acute Myocardial Infarction Complicated with Cardiogenic Shock (2021) [1]. (2) Patients with complete medical, examination, and surgical records.

#### 2.1.2. Exclusion Criteria

The exclusion criteria include the following: (1) Patients with acute blood disease, active autoimmune disease, or other diseases that may affect the prognosis of AMI. (2) Patients who discontinued treatment and were subsequently discharged. (3) Patients with incomplete data.

### 2.2. Data Collection

The patients’ general information, including demographic characteristics, vital signs upon admission, type of AMI, previous history of chronic medical conditions, complications, laboratory results (including arterial blood gas measurements, blood count, electrolyte levels, liver and kidney function, inflammatory factors, D‐dimer, and myocardial markers), and cardiac ultrasound results (including ejection fraction, left ventricular end size, and left atrial size) were obtained from the hospital’s electronic medical records, and the results of laboratory tests and cardiac ultrasound were recorded for the first time within 24 h of admission. Given that all‐cause death in the hospital was selected as the study endpoint, the number of days from onset to patient death was recorded, and the number of days from onset to patient recovery was recorded.

### 2.3. Statistical Analysis

SAS JMP10.0 was used for statistical analysis. The Kolmogorov‒Smirnov test was used to test the normality of the data. Normally distributed data are expressed as means and standard deviations, and differences between the two groups were compared using *t* tests. Non‐normally distributed data are expressed as medians and interquartile ranges, and differences between the two groups were compared using rank sum tests. Qualitative indicators are described as absolute numbers and component ratios, and the chi‐square test was used to compare differences between groups (the Fisher exact probability method or corrected chi‐square method was used if conditions were not met). With death or survival as the outcome, a ROC curve was drawn for the detection indicators, and the cutoff value was used for grouping in subsequent qualitative indicator analyses. A Kaplan‒Meier analysis was conducted according to the grouping to analyze the difference in survival time between different indicator groups. A Cox proportional hazards model was used to analyze the prognostic factors for the survival of AMICS patients. The detection indicators were first analyzed as quantitative indicators, and then the qualitative indicators were analyzed according to the group determined by the ROC curve. Cox analysis included unadjusted models, models adjusted for sex and age, and fully adjusted models. Factors in the fully adjusted model included sex, age, previous chronic history, severe complications, blood potassium level, urea level, creatinine level, albumin level, troponin level, BNP level, D‐dimer level, pH level, arterial bicarbonate ion level, arterial lactate level, procalcitonin level, EF value, left ventricular end size, and L/A. Based on the 20 variables included in the Cox regression analysis, the minimum sample size required is at least 300 people. A two‐tailed *p* value less than 0.05 indicated statistical significance.

## 3. Results

### 3.1. Comparison of the Clinical Features of Patients With AMICS

The clinical characteristics of 395 patients with AMICS were analyzed. The mean age of the patients was 67.03 ± 12.57 years, 277 males (70.13%), and 118 females (29.87%) were included, and 171 patients (43.29%) died. On average, the patients in the death group were older than those in the survival group were, and the proportion of females in the death group was significantly greater than that in the survival group (*p* < 0.05). In terms of vital signs at admission, diastolic blood pressure and heart rate were significantly higher in the death group than in the survival group (*p* < 0.05). The prevalence of prior myocardial infarction and Type 2 diabetes in the death group was significantly higher than that in the survival group (*p* < 0.05). Among the patients with severe complications of AMI, the incidence of atrial fibrillation was significantly higher in the death group than in the survival group (*p* < 0.05). The GRACE score of the death group was significantly higher than that of the survival group (*p* < 0.001). See Table 1 for details.

**TABLE 1:** Comparison of clinical features of AMICS patients [n (%)、 ($\overset{¯}{x}\pm s$)、M (Q_25_, Q_75_)].

Variables	Death group	Survival group	*Z*/*χ* ^2^	*p*
Age (years)	73.00 (61.00, 81.00)	64.00 (55.00, 73.00)	5.171	< 0.001
Sex				
Male (*n* (%))	111 (64.91)	166 (74.11)	3.914	0.048
Female (*n* (%))	60 (35.09)	58 (25.89)		
Smoking history				
Yes (*n* (%))	113 (66.08)	128 (57.14)	3.257	0.071
No (*n* (%))	58 (33.92)	96 (42.86)		
Admission vital signs				
SP (mmHg)	105.00 (86.00, 126.00)	108.00 (93.00, 126.00)	1.631	0.103
DP (mmHg)	64.30 ± 17.63	68.71 ± 14.97	2.622	0.009
HR (beats/min)	100.00 (76.25, 120.00)	90.00 (76.00, 110.00)	2.469	0.014
RR (beats/min)	21.00 (19.00, 27.75)	20.00 (18.00, 25.00)	1.768	0.077
STEMI				
Yes (*n* (%))	65 (38.01)	102 (45.54)	2.250	0.134
No (*n* (%))	106 (61.99)	122 (54.46)		
Past medical history				
OMI (*n* (%))	44 (25.73)	37 (16.52)	5.049	0.025
PCI (*n* (%))	35 (20.47)	36 (16.07)	1.271	0.260
CABG (*n* (%))	7 (4.09)	4 (1.79)	1.892	0.169^∗^
CKD (*n* (%))	23 (13.45)	21 (9.38)	1.627	0.202
Hypertension (*n* (%))	97 (56.73)	115 (51.34)	1.131	0.288
Type 2 diabetes (*n* (%))	79 (46.20)	75 (33.48)	6.593	0.010
Serious complication				
AF (*n* (%))	37 (21.64)	30 (13.39)	4.680	0.031
Perforation interventricular septum (*n* (%))	2 (1.17)	2 (0.89)	0.074	0.786
Left ventricular aneurysm (*n* (%))	14 (8.19)	22 (9.82)	0.313	0.576
VT/VF (*n* (%))	38 (22.22)	51 (22.77)	0.017	0.898
Third degree AVB (*n* (%))	12 (7.02)	15 (6.70)	0.016	0.900
Acute left heart failure (*n* (%))	166 (97.08)	222 (99.11)	2.306	0.129^∗^
Cardiac free wall rupture (*n* (%))	5 (2.92)	2 (0.89)	2.306	0.129^∗^
Mitral insufficiency (*n* (%))	12 (7.02)	15 (6.70)	0.016	0.900
GRACE score	187.00 (159.00, 210.00)	166.00 (145.00, 190.00)	5.014	< 0.001

*Note:* AVB, Atrioventricular block; DP, diastolic blood pressure; SP, systolic blood pressure; STEMI, acute ST elevation myocardial infarction.

Abbreviations: AF, atrial fibrillation; CABG, coronary artery bypass grafting; CKD, chronic kidney disease; HR, heart rate; OMI, old myocardial infarction; PCI, percutaneous coronary intervention; RR, respiratory rate; VT/VF, ventricular tachycardia/ventricular fibrillation.

^∗^Correction chi‐square.

### 3.2. Comparison of the Laboratory and Cardiac Ultrasound Results in Patients With AMICS

The laboratory and imaging indices of 395 patients with AMICS were analyzed, and the routine blood test results revealed that the platelet count in the death group was significantly lower than that in the survival group (*p* < 0.05). The biochemical results revealed that the intravenous serum bicarbonate level in the death group was lower than that in the survival group, and the serum potassium, sodium, urea, creatinine, total bilirubin, BNP, D‐dimer, procalcitonin, and IL‐6 levels were significantly higher than those in the survival group (*p* < 0.05).

Arterial blood gas analysis revealed that the arterial lactate level in the death group was greater than that in the survival group and that the pH and arterial bicarbonate ion levels were significantly lower than those in the survival group (*p* < 0.001).

Cardiac ultrasound revealed that the left ventricular end size and left atrial size in the death group were larger than those in the survival group, and the EF value was significantly lower than that in the survival group (*p* < 0.05).

The L/A in the death group was significantly greater than that in the survival group (*p* < 0.001). See Table 2 for details.

**TABLE 2:** Comparison of laboratory and cardiac ultrasound results in AMICS patients [M (Q_25_, Q_75_)].

Variables	Death group	Survival group	*Z*	*p*
Blood routine				
WBC (× 10^9^/L)	12.48 (8.92, 16.36)	11.22 (8.84, 14.73)	1.865	0.062
Hemoglobin (g/L)	123.00 (96.00, 139.00)	127.50 (110.00, 141.00)	1.931	0.054
PLT (× 10^9^/L)	203.00 (158.00, 254.00)	223.50 (174.25, 279.00)	2.565	0.010
Neutrophil count (× 10^9^/L)	10.62 (7.20, 13.47)	9.53 (6.76, 12.53)	1.451	0.147
Lymphocyte count (× 10^9^/L)	1.08 (0.66, 1.84)	1.12 (0.73, 1.54)	0.237	0.813
Monocyte count (× 10^9^/L)	0.69 (0.48, 1.00)	0.65 (0.43, 0.90)	1.528	0.127
Eosinophil count (× 10^9^/L)	0.00 (0.00, 0.03)	0.01 (0.00, 0.03)	0.717	0.473
Biochemical indices				
K^+^ (mmol/L)	4.10 (3.69, 4.84)	3.87 (3.56, 4.25)	3.757	< 0.001
Na^+^ (mmol/L)	139.53 (135.31, 142.36)	138.11 (135.76, 140.48)	2.181	0.029
Serum bicarbonate (mmol/L)	19.79 (16.63, 22.91)	22.51 (20.03, 25.29)	5.768	< 0.001
Urea (mmol/L)	10.15 (7.20, 17.27)	8.07 (6.00, 11.79)	3.723	< 0.001
Creatinine (μmol/L)	123.43 (80.96, 206.80)	83.81 (67.70, 121.39)	5.556	< 0.001
Albumin (g/L)	34.89 (30.24, 39.26)	34.41 (31.08, 37.83)	0.219	0.827
AST (U/L)	146.75 (47.39, 692.20)	173.68 (57.62, 495.23)	0.184	0.854
ALT (U/L)	58.00 (27.92, 211.00)	57.18 (33.65, 106.91)	0.328	0.743
Total bilirubin (μmol/L)	18.60 (13.29, 30.55)	17.57 (13.11, 23.44)	2.289	0.022
CK (IU/L)	416.52 (138.39, 1392.57)	695.24 (171.47, 2596.06)	1.729	0.084
CKMB (ng/mL)	13.20 (4.05, 50.60)	18.15 (4.00, 84.28)	1.230	0.219
Troponin I (ug/L)	9.69 (1.60, 56.30)	19.55 (2.99, 62.40)	1.864	0.062
NT‐proBNP (ng/L)	9510.00 (2580.00, 25, 500.00)	3880.00 (1230.00, 10, 675.00)	4.789	< 0.001
D‐dimer (ng/mL)	884.00 (297.50, 2747.00)	458.00 (209.00, 1238.50)	3.417	< 0.001
PCT (ng/mL)	0.28 (0.09, 1.40)	0.16 (0.06, 0.60)	2.981	0.003
IL‐6 (pg/mL)	61.59 (17.72, 193.70)	23.58 (11.45, 61.42)	4.778	< 0.001
CRP (mg/L)	34.95 (8.28, 75.75)	30.00 (11.65, 78.23)	0.284	0.776
Arterial blood gases				
PH	7.39 (7.27, 7.45)	7.42 (7.37, 7.47)	4.105	< 0.001
Bicarbonate ion (mmol/L)	16.20 (12.50, 18.80)	19.50 (17.15, 22.08)	7.064	< 0.001
Lactate (mmol/L)	2.90 (1.80, 7.40)	2.00 (1.50, 2.90)	6.100	< 0.001
Cardiac ultrasound				
EF (%)	43.56 (39.05, 53.58)	46.82 (40.95, 56.98)	2.737	0.006
Left ventricular (mm)	54.00 (50.00, 59.00)	52.00 (48.00, 57.00)	2.177	0.029
Left atrium (mm)	38.50 (35.00, 42.00)	36.00 (34.00, 40.00)	3.668	< 0.001
L/A	0.08 (0.05, 0.23)	0.06 (0.04, 0.09)	5.797	< 0.001

*Note:* ALT: alanine aminotransferase; AST: aspartate aminotransferase; PLT: platelet; NT‐proBNP: N‐terminal B natriuretic peptide precursor; IL‐6: Interleukin‐6; PCT: procalcitonin; L/A: lactate albumin ratio.

Abbreviations: CK, creatine kinase; CKMB, creatine kinase myocardial band; CRP, C‐reactive protein; EF, ejection fraction; WBC, white blood cell.

### 3.3. Predictive Value of Arterial Lactate Level, Arterial Bicarbonate Ion Level, Serum Bicarbonate Level, and the L/A for In‐Hospital Mortality in AMICS Patients

**FIGURE 1:**
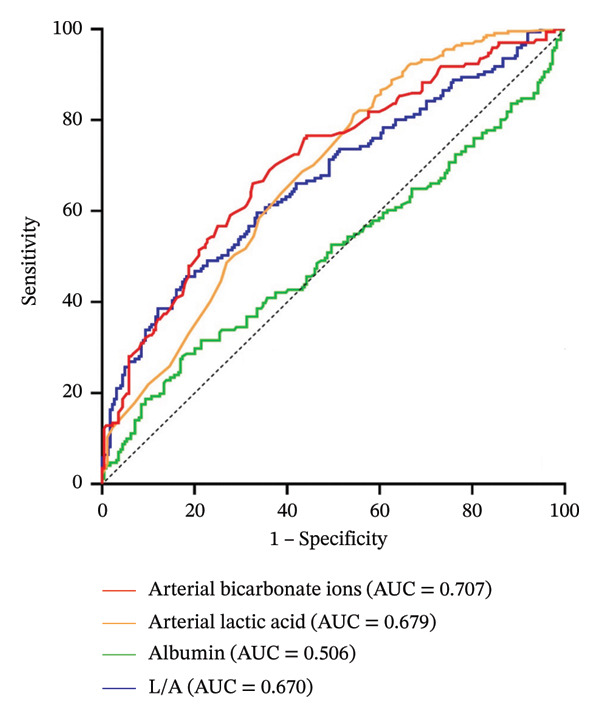
ROC curves for arterial bicarbonate ion level, arterial lactate levels, albumin level, and L/A for predicting hospital death in AMICS patients.

ROC curve analysis revealed that the areas under curves for arterial bicarbonate ion level, arterial lactate level, and L/A were 0.707, 0.679, and 0.670, respectively. The optimal cutoff value for the concentration of bicarbonate ions in arterial blood was 18.1, the sensitivity was 66.08%, and the specificity was 67.41%. The optimal cutoff value for arterial lactate level was 3.3, the sensitivity was 45.61%, and the specificity was 81.25%. The optimal cutoff value for L/A was 0.1015, the sensitivity was 45.61%, and the specificity was 91.70%. The areas under the curve for the above three indicators were all greater than 0.65, indicating that they have certain predictive value for in‐hospital death in AMICS patients. The arterial bicarbonate ion level had the highest predictive value, followed by arterial blood lactic acid and L/A. The area under the curve for the albumin level was 0.506, and the optimal cutoff value was 26.88, indicating that the albumin level could not be used to predict in‐hospital death in AMICS patients. See Figure 1 for details.

### 3.4. Kaplan–Meier Survival Curves for AMICS Patients

**FIGURE 2:**
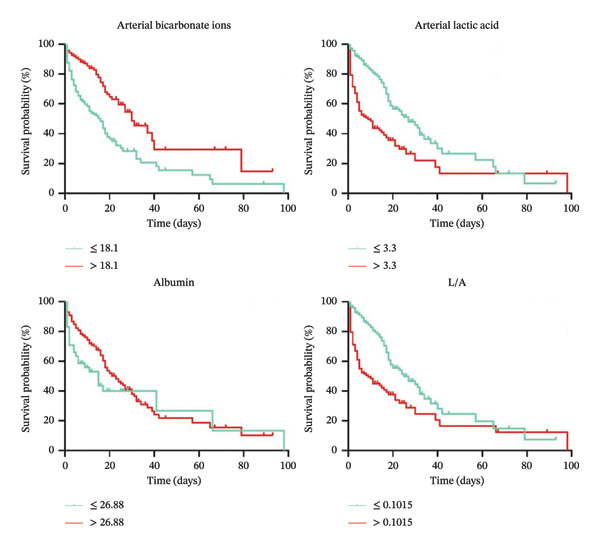
Kaplan‒Meier survival curves for AMICS patients.

Subgroup survival curves were analyzed using the optimal cutoff values for arterial bicarbonate ion level, arterial lactate level, albumin level, and L/A to predict in‐hospital death in AMICS patients. Figure 2 shows that the median survival time of the arterial bicarbonate ion ≤ 18.1 group was 16 days and that of the arterial bicarbonate ion > 18.1 group was 30 days (log‐rank test: *χ* ^2^ = 40.460, *p* < 0.001). The median survival time of the lactic acid ≤ v3.3 group was 27 days and that of the lactic acid > 3.3 group was 9 days (log‐rank test: *χ* ^2^ = 65.810, *p* < 0.001). The median survival time of the albumin ≤ 26.88 group was 15 days and that of the albumin > 26.88 group was 23 days (log‐rank test: *χ* ^2^ = 9.161, *p* = 0.003). The median survival time of the L/A ≤ 0.1015 group was 25 days and that of the L/A > 0.1015 group was 9 days (log‐rank test: *χ* ^2^ = 63.690, *p* < 0.001). In conclusion, a decrease in the arterial bicarbonate ion level, a decrease in the albumin level, an increase in the arterial lactate level, and an increase in L/A suggest an increased risk of in‐hospital mortality.

### 3.5. Cox Proportional Risk Model for In‐Hospital Death in AMICS Patients

With AMICS (death or survival) as the dependent variable and death (*Y* = 1) as the event, factors that may have a greater impact on survival time among the variables were selected as independent variables according to clinical experience, combined with the results of univariate analysis, and included in the Cox proportional risk model for analysis. Table 3 shows that a higher arterial bicarbonate ion level is a protective factor for in‐hospital death in AMICS patients. A higher arterial lactate level and a higher L/A were found to be independent risk factors for in‐hospital death in AMICS patients.

**TABLE 3:** Cox proportional risk model for in‐hospital death in AMICS patients.

Variables	Mortality rate (%)	Unadjusted		Adjust for age and sex		Complete adjustment	
		HR (95% CI)	*p*	HR (95% CI)	*p*	HR (95% CI)	*p*
Arterial bicarbonate ion (quantitative)	171/395 (43.29)	0.901 (0.875–0.928)	< 0.001	0.903 (0.876–0.930)	< 0.001	0.764 (0.675–0.859)	< 0.001
Arterial bicarbonate ion (qualitative)							
≤ 18.1 mmol/L	113/186 (60.75)	1		1		1	0.039
> 18.1 mmol/L	40/209 (19.14)	0.403 (0.235–0.554)	< 0.001	0.414 (0.301–0.570)	< 0.001	0.395 (0.163–0.955)	
Arterial lactate (quantitative)	171/395 (43.29)	1.152 (1.113–1.191)	< 0.001	7.822 (4.759–12.538)	< 0.001	1.438 (1.235–1.676)	< 0.001
Arterial lactate (qualitative)							
≤ 3.3 mmol/L	95/279 (34.05)	1		1		1	0.001
> 3.3 mmol/L	76/116 (65.52)	2.473 (1.823–3.353)	< 0.001	2.583 (1.901–3.508)	< 0.001	5.754 (1.927–17.180)	
Albumin (quantitative)	171/395 (43.29)	1.013 (0.985–1.042)	0.377	2.093 (0.777–5.773)	0.149	1.133 (1.045–1.302)	0.065
Albumin (qualitative)							
≤ 26.88 g/L	26/41 (63.41)	1		1		1	0.110
> 26.88 g/L	145/354 (40.96)	0.696 (0.454–1.067)	0.096	0.787 (0.511–1.212)	0.277	0.207 (0.029–1.463)	
L/A (quantitative)	171/395 (43.29)	1.359 (1.242–1.477)	< 0.001	1.397 (1.274–1.521)	< 0.001	1.224 (1.093–1.362)	< 0.001
L/A (qualitative)							
≤ 0.1015	94/277 (33.94)	1		1		1	0.005
> 0.1015	77/118 (65.25)	2.330 (1.718–3.160)	< 0.001	2.310 (1.703–3.134)	< 0.001	2.171 (1.667–16.046)	

When the measurement indices were entered into the equation as quantitative data, arterial bicarbonate ion level, lactate level, and L/A all had certain effects on in‐hospital death in different adjusted models. According to the fully adjusted analysis, the risk of in‐hospital death increased by 43.8% for each 1 mmol/L increase in arterial blood lactic acid. For every 0.1 increase in L/A, the risk of in‐hospital death increased by 22.4%. The risk of in‐hospital death decreased by 23.6% for every 1 mmol/L increase in the arterial bicarbonate ion level.

Qualitative analysis was conducted after the patients were grouped according to the optimal cutoff value, and the risk of in‐hospital death in patients with an arterial lactate level > 3.3 was 5.754 times greater than that in patients with an arterial lactate level ≤ 3.3. The risk of in‐hospital death in patients with a L/A > 0.1015 was 2.171 times greater than that in patients with a L/A ≤ 0.1015. The risk of in‐hospital death in patients with an arterial blood bicarbonate concentration > 18.1 was 0.395 times greater than that in patients with an arterial blood bicarbonate ion concentration ≤ 18.1. The power value for arterial blood bicarbonate concentration is 0.926, and the power values for the arterial lactate level and L/A are both greater than 0.99.

## 4. Discussion

After AMICS has been clearly diagnosed, early and accurate assessment of the disease and early appropriate comprehensive supportive treatment may reduce the mortality rate and save more lives.

In this study, 395 AMICS patients admitted to a large hospital in Xinjiang over the past five years were included. The predictive value of early arterial lactate levels, arterial bicarbonate ion levels, and the L/A for in‐hospital mortality in AMICS patients was retrospectively analyzed, and it was found that the arterial bicarbonate ion level had the highest predictive efficacy, followed by arterial lactate levels and L/A. These findings are slightly different from those of several previous studies. In an analysis based on the MIMIC public database, Zhu et al. [22], Chen et al. [23], and Wang et al. [24] reported that L/A was better than lactate levels or albumin alone in predicting the prognosis of AMI patients. However, in this study, the ROC curve analysis indicated that the serum concentration alone had no predictive value. While L/A alone has the same predictive value as the lactic acid level alone, their combined use does not show obvious advantages over the use of either index alone, possibly because only critically ill AMI patients were included in other studies, as was a broad definition of critical illness not necessarily accompanied by CS. However, all the participants included in this study were patients with a definite diagnosis of AMICS, which may be the main reason for the difference in research results. Moreover, the patient data in the MIMIC public database used by the above three researchers were mainly from the U.S. population, while the people included in this study were from the Xinjiang region of China. The differences in region may also be one of the reasons for the differences in research results.

In patients with AMICS, ischemia, hypoxia, and mitochondrial dysfunction in tissues increase anaerobic glycolysis. Lactic acid, the main product of anaerobic glycolysis, can reflect the oxygen supply and perfusion level of tissues, as well as the severity and prognosis of AMICS [25]. According to the current expert consensus [1], arterial lactate level is a recommended indicator for predicting the prognosis of AMICS as well as evaluating the efficacy of treatment for AMICS and arterial lactate > 6.5 mmol/L is an important independent predictor of an increased risk of in‐hospital mortality in AMICS patients [26]. Gjesdal [27] conducted a study on a Swedish population and reported that arterial lactate ≥ 2.5 mmol/L was associated with 30‐day mortality in Killip Grade II‐III AMI patients. Vermeulen et al.[11] reported that acute mortality in STEMI patients was correlated with arterial lactate ≥ 1.8 mmol/L at hospital admission. In this study, univariate analysis revealed that the arterial lactate levels and L/A were greater in the death group than in the survival group, which was consistent with the findings of previous studies [11, 12, 26, 27]. Moreover, the K–M survival curve revealed that arterial lactate > 3.3 mmol/L significantly reduced the median survival time and significantly increased the risk of in‐hospital death. The Cox proportional risk model showed that the risk of in‐hospital death increased by 43.8% for every 1 mmol/L increase in arterial lactate. This level is lower than the lactate level recommended by expert consensus but higher than the lactate level proposed by other researchers, mainly because of differences in the participants selected. However, the overall results of this and other studies indicate that the higher the early arterial lactate level is, the higher the AMI (or CS)‐related mortality rate.

Albumin is a negatively charged reactive protein whose main physiological functions include regulating plasma osmotic pressure, providing energy for oxidative decomposition under severe consumption, maintaining the acid‒base balance and antioxidation [28]. However, in cardiovascular diseases, González‐Pacheco [29] reported that low albumin levels were independent predictors of new heart failure and in‐hospital mortality in patients with acute coronary syndrome. In this study, for the AMICS population in Xinjiang, China, univariate analysis revealed that there was no significant difference in the albumin level between the death group and the survival group, and ROC curve analysis also revealed that the area under the curve for the albumin level was 0.506, indicating that the albumin level could not be used to predict in‐hospital mortality in AMICS patients. Only the K‒M survival curve revealed that the median survival time of the albumin level ≤ 26.88 group was significantly lower than that of the albumin level > 26.88 group. In the Cox‐fitted proportional risk model, neither quantitative nor qualitative analysis yielded a positive result for the albumin level. Therefore, the albumin level alone is not a predictor of prognosis in AMICS patients.

The L/A is a complex indicator that represents both inflammation and nutrition status. In this study, although the ROC curve analysis suggested that the L/A had a modest predictive value for in‐hospital death in AMICS patients, the K‒M survival curve still revealed that the median survival time of patients with a L/A ≤ 0.1015 was significantly extended and that an increase in L/A suggested an increased risk of in‐hospital death. The Cox proportional risk model showed that for every 0.1 increase in L/A, the risk of in‐hospital death increased by 22.4%, and the risk of in‐hospital death in patients with L/A > 0.1015 was 2.171 times greater than that in patients with L/A ≤ 0.1015. This finding is similar to that of previous studies. In a study involving people with severe AMI, Yang [23] suggested that the risk of in‐hospital death in the L/A ≥ 0.709 group was higher than that in the L/A < 0.709 group. Zhu et al. [22] suggested that the risk of 30‐day all‐cause death in the L/A ≤ 0.97 group and the L/A > 0.97 group was higher than that in the L/A ≤ 0.47 group. The K‒M survival curve in Wang et al.’s [24] study revealed that patients with a L/A > 0.6667 had the highest mortality rate, and the multivariate Cox proportional risk model verified that the L/A was significantly correlated with 14‐day, 28‐day, and 90‐day all‐cause mortality in critically ill AMI patients. Compared with that reached in previous studies, the conclusion reached in this study was that a higher L/A is an independent risk factor for in‐hospital death in AMI patients, possibly because of differences in the cutoff values, thus further exploration in large‐sample studies is needed.

The arterial bicarbonate ion concentration is also an important indicator of the acid‒base balance in the human body. In this study, univariate analysis revealed that the arterial bicarbonate ion concentration in the death group was significantly lower than that in the survival group. ROC curve analysis revealed that arterial bicarbonate ion had a higher predictive value for in‐hospital death in AMICS patients, even higher than the L/A composite index. The K‒M survival curve also revealed that when the arterial bicarbonate ion concentration was ≤ 18.1 mmol/L, the risk of in‐hospital mortality increased. The Cox proportional hazards model revealed that the risk of in‐hospital death decreased by 23.6% for every 1 mmol/L increase in arterial bicarbonate ion concentration. This finding is in line with previous findings: Wigger et al. [13] conducted a large cohort study of CS patients consecutively admitted to the ICU and reported that baseline serum bicarbonate levels independently predicted 28‐day mortality in patients with CS. Zhu et al.[22] reported that when the bicarbonate ion concentration was less than 22 mmol/L, the 30‐day risk of mortality in patients with severe AMI increased with decreasing bicarbonate concentration. The risk of death was lowest when the concentration was in the range of 22–27 mmol/L. Therefore, higher arterial bicarbonate ion levels are a protective factor for in‐hospital death in AMICS patients.

In AMICS, lactate serves as a product of both systemic hypoperfusion and microcirculatory dysfunction. Elevated lactate levels indicate persistent tissue hypoxia, acting as an initiating signal for cellular shock and multiple organ failure. The reduction in arterial bicarbonate not only represents a metabolic consequence of acidosis but also reflects the depletion of the body’s buffering capacity. Severe acidosis can suppress myocardial contractility, diminish vascular responsiveness to vasoactive agents, and trigger arrhythmias, thereby establishing a vicious cycle. An elevated L/A ratio—driven by increased lactate (numerator) and decreased albumin (denominator)—signifies a dual insult of “high injury drive” coupled with “low endogenous protection.” This integrated parameter offers a more comprehensive reflection of the patient’s overall critical condition and exhaustion of physiological reserve than any single indicator alone.

In summary, our study revealed that early arterial bicarbonate ion levels, arterial lactate levels, and the L/A are reliable predictors of all‐cause in‐hospital death in AMICS patients. Early measurement of arterial blood gases and albumin levels, particularly at admission, is recommended for faster and more convenient assessment of mortality risk in AMICS patients as well as for timely and optimal medical management.

This study has notable limitations [1]. As a single‐center retrospective study, the use of only one database and a small sample of patients who were all from the same region and of the same ethnicity limited the generalizability of the study findings [2]. In this study, only the arterial blood gases and albumin levels measured at admission were recorded and analyzed, and subsequent dynamic changes were not recorded [3]. To directly assess overfitting, we performed bootstrap internal validation with 1000 repetitions. The results indicated a modest degree of optimism (0.07), which reduced the C‐index from 0.78 to 0.71 after correction. Nevertheless, this level of optimism remains within an acceptable range for moderate‐dimensional clinical prediction models. The corrected C‐index (0.71) still reflects meaningful predictive discrimination. Further external validation in a larger independent cohort will be conducted to confirm the model’s robustness. In the future, we plan to use more data from more patients and conduct trajectory analyses to ensure the accuracy of the study.

## Author Contributions

Jiao Wang and Meijuan Zheng collected the data, analyzed and interpreted patient data, and were major contributors to writing the manuscript. Yuchun Yang and Zhen Bao collected the data and participated in editing the manuscript. Muhuyati Wulasihan contributed to the conception and design of this study and editing of the manuscript.

## Funding

This work was supported by the funding from the Key Laboratory of Special Environment and Health Research in Xinjiang (grant No. SKL‐SEHR‐2024‐04), the Natural Science Foundation of Xinjiang Uygur Autonomous Region—Young Scientists Fund (Grant No. 2025D01C428), and the National Natural Science Foundation of China (Grant No. 82560064).

## Disclosure

All authors read and approved the final manuscript.

## Ethics Statement

The retrospective study was approved by the Medical Ethics Committee of the First Affiliated Hospital of Xinjiang Medical University (approval No. K202412‐25) and was conducted in accordance with the Declaration of Helsinki and its amendments.

## Conflicts of Interest

The authors declare no conflicts of interest.

## Data Availability

The data that support the findings of this study are available from the corresponding author upon reasonable request.

## References

[1] Chinese Society of Cardiology, Editorial Board of Chinese Journal of Cardiology, Chinese Expert Consensus on Diagnosis and Treatment of Acute Myocardial Infarction Combined With Cardiogenic Shock. (2021) 50, 231–242, Chinese Journal of Cardiovascular Diseases. 10.3760/cma.j.cn112148-20210706-00574 35340141

[2] Song F. , Yu M. , Yang J. et al., Symptom-Onset-to-Balloon Time, ST-Segment Resolution and In-Hospital Mortality in Patients With ST-Segment Elevation Myocardial Infarction Undergoing Primary Percutaneous Coronary Intervention in China: From China Acute Myocardial Infarction Registry, American Journal of Cardiology. (2016) 118, no. 9, 1334–1339, 10.1016/j.amjcard.2016.07.058, 2-s2.0-84992488265. 27666173

[3] De Luca L. , Olivari Z. , Farina A. et al., Temporal Trends in the Epidemiology, Management, and Outcome of Patients With Cardiogenic Shock Complicating Acute Coronary Syndromes, European Journal of Heart Failure. (2015) 17, no. 11, 1124–1132, 10.1002/ejhf.339, 2-s2.0-84955173380. 26339723

[4] Scholz K. H. , Maier S. K. G. , Maier L. S. et al., Impact of Treatment Delay on Mortality in ST-segment Elevation Myocardial Infarction (STEMI) Patients Presenting With and Without Haemodynamic Instability: Results From the German Prospective, Multicentre FITT-STEMI Trial, European Heart Journal. (2018) 39, no. 13, 1065–1074, 10.1093/eurheartj/ehy004, 2-s2.0-85045463809. 29452351 PMC6018916

[5] National Cardiovascular Center , China Cardiovascular Health and Disease Report Compilation Group, Shengshou Hu. Summary of China Cardiovascular Health and Disease Report 2023, Chinese Journal of Circulation.(2024) 39, 625–660.

[6] Auffret V. , Cottin Y. , Leurent G. et al., Predicting the Development of In-Hospital Cardiogenic Shock in Patients With ST-Segment Elevation Myocardial Infarction Treated by Primary Percutaneous Coronary Intervention: The ORBI Risk Score, European Heart Journal. (2018) 39, no. 22, 2090–2102, 10.1093/eurheartj/ehy127, 2-s2.0-85048668008. 29554243

[7] Vincent J. L. , Quintairos E. , Silva A. , Couto L. , and Taccone F. S. , The Value of Blood Lactate Kinetics in Critically Ill Patients: A Systematic Review, Critical Care. (2016) 20, no. 1, 10.1186/s13054-016-1403-5, 2-s2.0-85008627543. PMC4983759 27520452

[8] Nichol A. D. , Egi M. , Pettila V. et al., Relative Hyperlactatemia and Hospital Mortality in Critically Ill Patients: A Retrospective Multi-Centre Study, Critical Care. (2010) 14, no. 1, 10.1186/cc8888, 2-s2.0-77953000739. PMC2875540 20181242

[9] Zymliński R. , Biegus J. , Sokolski M. et al., Increased Blood Lactate is Prevalent and Identifies Poor Prognosis in Patients With Acute Heart Failure Without Overt Peripheral Hypoperfusion, European Journal of Heart Failure. (2018) 20, no. 6, 1011–1018, 10.1002/ejhf.1156, 2-s2.0-85041907580. 29431284

[10] Williams T. A. , Martin R. , Celenza A. et al., Use of Serum Lactate Levels to Predict Survival for Patients With Out-of-Hospital Cardiac Arrest: A Cohort Study, Emergency Medicine Australasia. (2016) 28, no. 2, 171–178, 10.1111/1742-6723.12560, 2-s2.0-84959277392. 26929190

[11] Vermeulen R. P. , Hoekstra M. , Nijsten M. W. et al., Clinical Correlates of Arterial Lactate Levels in Patients With ST-Segment Elevation Myocardial Infarction at Admission: A Descriptive Study, Critical Care. (2010) 14, no. 5, 10.1186/cc9253, 2-s2.0-77956460366. PMC3219257 20825687

[12] Hayıroğlu M. İ. , Keskin M. , Uzun A. O. et al., Predictors of In-Hospital Mortality in Patients With ST-Segment Elevation Myocardial Infarction Complicated With Cardiogenic Shock, Heart Lung & Circulation. (2019) 28, no. 2, 237–244, 10.1016/j.hlc.2017.10.023, 2-s2.0-85035222564. 29191504

[13] Wigger O. , Bloechlinger S. , Berger D. et al., Baseline Serum Bicarbonate Levels Independently Predict Short-Term Mortality in Critically Ill Patients With Ischaemic Cardiogenic Shock, European Heart Journal–Acute Cardiovascular Care. (2018) 7, no. 1, 45–52, 10.1177/2048872616683526, 2-s2.0-85051047297. 28838261

[14] Tan L. , Xu Q. , Li C. , Chen X. , and Bai H. , Association Between the Admission Serum Bicarbonate and Short-Term and Long-Term Mortality in Acute Aortic Dissection Patients Admitted to the Intensive Care Unit, International Journal of General Medicine. (2021) 14, 4183–4195, 10.2147/ijgm.s321581. 34385839 PMC8352635

[15] He Y. and Yang X. , Case-Control Study on Screening Risk Factors of Atrial Fibrillation, Chinese Circulation Journal. (2005) 132, 114–117.

[16] He Y. M. , Yang X. J. , Hui J. et al., Low Serum Albumin Levels in Patients With Paroxysmal Atrial Fibrillation: What Does it Mean?, Acta Cardiologica. (2006) 61, no. 3, 333–337, 10.2143/ac.61.3.2014837, 2-s2.0-33745748743. 16869456

[17] He Y. M. , Yang Q. , Yang X. J. , Zhao X. , Xu H. F. , and Qian Y. X. , Serum Albumin Concentrations, Effect Modifiers and First Incident Acute Myocardial Infarction: A Cross-Sectional Study of 1552 Cases and 6680 Controls, Clinica Chimica Acta. (2016) 454, 49–56, 10.1016/j.cca.2015.12.037, 2-s2.0-84953262658. 26747960

[18] Xia M. , Gu J. , Zhang C. , He Y. , and Yang X. , Correlation Between Serum Albumin Level and Primary Acute Myocardial Infarction, Chinese Journal of Geriatric Cardio-Cerebrovascular Diseases.(2018) 20, 153–157.

[19] Shin J. , Hwang S. Y. , Jo I. J. et al., Prognostic Value of the Lactate/Albumin Ratio for Predicting 28-Day Mortality in Critically ILL Sepsis Patients, Shock. (2018) 50, no. 5, 545–550, 10.1097/shk.0000000000001128, 2-s2.0-85054896883. 29461463

[20] Cakir E. and Turan I. O. , Lactate/Albumin Ratio is More Effective Than Lactate or Albumin Alone in Predicting Clinical Outcomes in Intensive Care Patients With Sepsis, Scandinavian Journal of Clinical & Laboratory Investigation. (2021) 81, no. 3, 225–229, 10.1080/00365513.2021.1901306. 33745405

[21] Guo W. , Zhao L. , Zhao H. , Zeng F. , Peng C. , and Yan H. , The Value of Lactate/Albumin Ratio for Predicting the Clinical Outcomes of Critically Ill Patients With Heart Failure, Annals of Translational Medicine. (2021) 9, no. 2, 10.21037/atm-20-4519. PMC7867948 33569420

[22] Zhu J. L. , Liu H. , Wang L. L. et al., Association of Lactate to Albumin Ratio and Bicarbonate With Short-Term Mortality Risk in Patients With Acute Myocardial Infarction, BMC Cardiovascular Disorders. (2022) 22, no. 1, 10.1186/s12872-022-02902-4. PMC9673455 36401181

[23] Chen Y. , Lai W. , Yang K. , Wu B. , Xie D. , and Peng C. , Association Between Lactate/Albumin Ratio and Prognosis in Patients With Acute Myocardial Infarction, European Journal of Clinical Investigation. (2024) 54, no. 1, 10.1111/eci.14094. 37725487

[24] Wang D. , Luo C. , Li Q. et al., Association Between Lactate/Albumin Ratio and All-Cause Mortality in Critical Patients With Acute Myocardial Infarction, Scientific Reports. (2023) 13, no. 1, 10.1038/s41598-023-42330-8. PMC10511737 37730950

[25] Kubiak G. M. , Tomasik A. R. , Bartus K. , Olszanecki R. , and Ceranowicz P. , Lactate in Cardiogenic Shock-Current Understanding and Clinical Implications, Journal of Physiology & Pharmacology. (2018) 69, no. 1, 15–21, 10.26402/jpp.2018.1.02, 2-s2.0-85046424743. 29769417

[26] Li C. L. , Wang H. , Jia M. , Meng X. , and Hou X. T. , The Early Dynamic Behavior of Lactate is Linked to Mortality in Postcardiotomy Patients With Extracorporeal Membrane Oxygenation Support: A Retrospective Observational Study, Journal of Thoracic and Cardiovascular Surgery. (2015) 149, no. 5, 1445–1450, 10.1016/j.jtcvs.2014.11.052, 2-s2.0-84929607084. 25534305

[27] Gjesdal G. , Braun O. Ö. , Smith J. G. , Scherstén F. , and Tydén P. , Blood Lactate is a Predictor of Short-Term Mortality in Patients With Myocardial Infarction Complicated by Heart Failure but Without Cardiogenic Shock, BMC Cardiovascular Disorders. (2018) 18, no. 1, 10.1186/s12872-018-0744-1, 2-s2.0-85040738490. PMC5774118 29347907

[28] Chen C. B. , Hammo B. , Barry J. , and Radhakrishnan K. , Overview of Albumin Physiology and Its Role in Pediatric Diseases, Current Gastroenterology Reports. (2021) 23, no. 8, 10.1007/s11894-021-00813-6. 34213692

[29] González-Pacheco H. , Amezcua-Guerra L. M. , Sandoval J. et al., Prognostic Implications of Serum Albumin Levels in Patients With Acute Coronary Syndromes, American Journal of Cardiology. (2017) 119, no. 7, 951–958, 10.1016/j.amjcard.2016.11.054, 2-s2.0-85011092570. 28160977

